# Selective Recovery of Metallic Zinc from Zinc Leaching Residue by Calcification Roasting and Acid Leaching

**DOI:** 10.3390/ma18040738

**Published:** 2025-02-07

**Authors:** Zhenqi Wang, Hui Ge, Feng Xie, Shaohua Wu, Wang Wei

**Affiliations:** 1 Key Laboratory for Ecological Metallurgy of Multimetallic Ores (Ministry of Education), Northeastern University, Shenyang 110819, China; clyy_530@163.com (Z.W.); wushfly@163.com (S.W.); wangwei@smm.neu.edu.cn (W.W.); 2 School of Metallurgy, Northeastern University, Shenyang 110819, China; 3 Zijin Mining Group Co., Ltd., Longyan 364200, China

**Keywords:** zinc leaching residue, calcification roasting, acid leaching, zinc

## Abstract

It is essential to recycle zinc leaching residue (ZLR) generated by the conventional zinc hydrometallurgy process, as it is a hazardous and potentially valuable industrial waste. A combined calcification roasting–acid leaching process was developed to selectively separate and recover zinc from ZLR. This work investigates the effectiveness of using calcium oxide as an additive to transform zinc ferrite during the roasting process. The feasibility of the reaction was investigated based on thermodynamic calculations and compositional analysis. The transformation ratio of zinc ferrite reached 95.27% after roasting at 900 °C for 2 h with a Ca/Fe molar ratio of 3. During the calcification roasting process, the zinc ferrite was effectively converted into zinc oxide and calcium ferrite. The selective leaching of zinc was achieved at an L/S of 15, 25 g/L H_2_SO_4_, 60 °C, and 90 min. The extraction ratios of Zn and Fe were 86.26% and 0.06%, respectively. After the leachate was evaporated and purified, metallic zinc with a purity of 99.53% was obtained by constant current electrolysis for 60 min with a current efficiency of 86.7%. The proposed process provides a viable alternative method for recycling zinc resources from ZLR by an environmentally friendly method.

## 1. Introduction

Currently, approximately 85% of zinc was produced by using the roasting–leaching–purification–electrowinning process [1,2,3]. In the oxidation roasting of sphalerite, a fraction of the zinc combines with iron impurities to generate zinc ferrite [4]. Zinc ferrite (ZnFe_2_O_4_) was a ferrite of spinel type, possessing a remarkably stable crystal structure that remains insoluble in conventional sulfuric acid leaching processes [5]. This feature results in the enrichment of zinc ferrite in ZLR (0.5–0.9 tons per ton of zinc), which contains zinc, iron, lead, and other metals [6,7]. However, it was important to note that ZLR is classified as hazardous waste due to the presence of heavy metals [8,9]. Therefore, the accumulation and storage of ZLR have caused serious pollution to the surrounding environment [10]. Given these circumstances, the implementation of a recycling process for ZLR is imperative to effectively address the environmental pollution issue.

The depletion of natural resources has made resource recycling a crucial issue to consider in future development trends. The ZLR was usually recycled by hydrometallurgical processes, pyrometallurgical processes, and their combination. A variety of hydrometallurgical processes have been developed, including acid leaching, alkaline leaching, brine leaching, and high-pressure acid leaching [11,12,13]. The acid leaching process is the most widely used and requires high temperatures (90–95 °C) and high concentrations of acid (200–250 g/L) to achieve the dissolution of zinc ferrite in the ZLR, as shown in Equation (1). Most of the iron in ZLR is extracted into the leaching solution, which complicates the purification of the leachate solution. Additionally, there were many pyrometallurgical processes for ZLR recovery, among which the Waelz process is the most typical and has been used in industrial production [14,15,16,17,18]. The Waelz process requires significant energy consumption to maintain the high reaction temperatures (1100–1300 °C) and the selective volatilization of zinc, alkali metals, and their compounds from the furnace charge [19]. Excessive energy consumption, high product impurities, and the generation of a large number of secondary residues with high iron content have limited the development of the process [20,21]. Some new processes combining pyrometallurgical and hydrometallurgical processes were developed, such as the reduction roasting–acid leaching process [3,4,14], Na_2_CO_3_ roasting–acid leaching process [22], and sulfate roasting–acid leaching process [23]. The roasting process in the above processes destroys the structure of zinc ferrite in ZLR and converts it into a metal salt that can be easily extracted.

ZnFe_2_O_4_ + 4H_2_SO_4_ = ZnSO_4_ + Fe_2_(SO_4_)_3_ + 4H_2_O (1)

In this work, calcium oxide is utilized as an additive to transform zinc ferrite. The objective is to evaluate the potential of using calcium oxide as an additive for recovering metal values from ZLR. The leaching conditions were then controlled to achieve the separation of Zn and Fe. Compared to the conventional pyrometallurgical process for treating ZLR, calcification roasting consumes less energy and does not generate sulfur-containing soot, which is more environmentally friendly. The feasibility of the reaction was investigated based on thermodynamic calculations and compositional analysis. The transformation of the mineral phase and the evolution of the microstructure in the roasted products were extensively studied using techniques such as X-ray diffraction (XRD), scanning electron microscopy (SEM), and energy dispersive spectrometry (EDS) to identify the possible mechanisms involved in the calcification roasting process.

## 2. Experiment

### 2.1. Raw Materials

**Figure 1:**
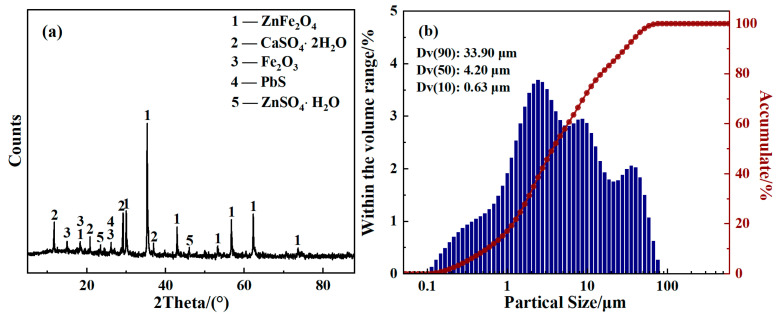
Characterizations of the ZLR sample: (**a**) XRD pattern; (**b**) particle size distribution.

The ZLR sample taken from a zinc hydrometallurgical plant in the Inner Mongolia Autonomous Region of China was dried at 100 °C for 48 h, and then ground and sieved. The XRD pattern reveals that zinc ferrite (ZnFe_2_O_4_), calcium sulfate (CaSO_4_·2H_2_O), ferric oxide (Fe_2_O_3_), lead sulfide (PbS), and zinc sulfate (ZnSO_4_·H_2_O) were the major phases in the ZLR as shown in Figure 1a. The particle size distribution analysis of the ZLR sample with a laser particle size analyzer is shown in Figure 1b. As shown in the cumulative volume content results, ZLR has a fine particle size with a D50 value of 4.20 μm. The calcium oxide and sulfuric acid used in the experiments were analytical grade reagents (Sinopharm Chemical Reagent Co., Ltd., Shanghai, China).

### 2.2. Experimental Procedure

#### 2.2.1. Calcification Roasting

The temperature of the reaction was adjusted between 700 °C and 1100 °C, using a step of 100 °C. The duration of roasting was between 0.5 h and 2.5 h, with increments of 0.5 h. The molar ratio of Ca to Fe was used to indicate the amount of CaO added, with the Ca/Fe molar ratio ranging from 1 to 5. The dried ZLR was thoroughly mixed with a certain amount of CaO and pressed at a pressure of about 10 MPa for 10 min to form a uniform cylindrical briquette with a diameter of 10 mm and a height of 10 mm. These cylindrical briquettes were then placed into a corundum crucible and heated at a specific temperature in a muffle furnace. Once roasted, the samples were taken out from the muffle furnace, cooled in the air, and analyzed. The 5 g of roasted sample was added to 150 mL of H_2_SO_4_ solution with a concentration of 100 g/L and leached at 60 °C for 90 min. The effect of calcification roasting was measured by the extraction ratio of zinc. The transformation ratio of zinc ferrite was calculated according to Equation (2).

(2) $$R=\frac{C_{Zn}\;\text{×}\;V}{W_{Zn}\;\text{×}\;m}\;\text{×}\;100\%$$

where *R* is the transformation ratio of zinc ferrite, %; *C_Zn_* is the concentration of Zn in leachate, g/L; *V* is the volume of the leachate, L; *W_Zn_* is the content of Zn or Fe in the roasted ZLR sample, %; *m* is the mass of the roasted ZLR sample, g.

#### 2.2.2. Acid Leaching

Sulfuric acid leaching was a process used to dissolve soluble substances in the roasted product and create an aqueous solution. To investigate the impact of acid concentration, leaching temperature, liquid-to-solid ratio (L/S), and leaching time on the extraction ratios of Zn and Fe, experiments were carried out. The liquids used in the experiment were measured using measuring cylinders and volumetric flasks. The roasted ZLR was combined with a sulfuric acid solution ranging from 25 g/L to 125 g/L, with an L/S varying from 5 to 25. The leaching process was conducted at temperatures between 40 °C and 80 °C, using a water bath, for different durations ranging from 15 min to 120 min. Throughout the experiment, the suspension was continuously stirred at a fixed speed of 400 rpm using a magnetic stirrer. Periodically, 2 mL of filtrate samples were taken out by a microporous filter, and the concentration of Zn and Fe were determined by the inductively coupled plasma–atomic emission spectroscopy method. Afterward, the leaching slurry was separated by a vacuum filter. The solid residues were washed and dried, and then the solid residue composition was determined by XRD. The extraction ratios of Zn and Fe were calculated according to Equation (3).

(3) $$E=\frac{C\;\text{×}\;V}{W\;\text{×}\;m}\;\text{×}\;100\%$$

where *E* is the extraction ratio of Zn or Fe, %; *C* is the concentration of Zn or Fe in leachate, g/L; *V* is the volume of the leachate, L; *W* is the content of Zn or Fe in the roasted ZLR sample, %; *m* is the mass of the roasted ZLR sample, g.

#### 2.2.3. Electrowinning

**Figure 2:**
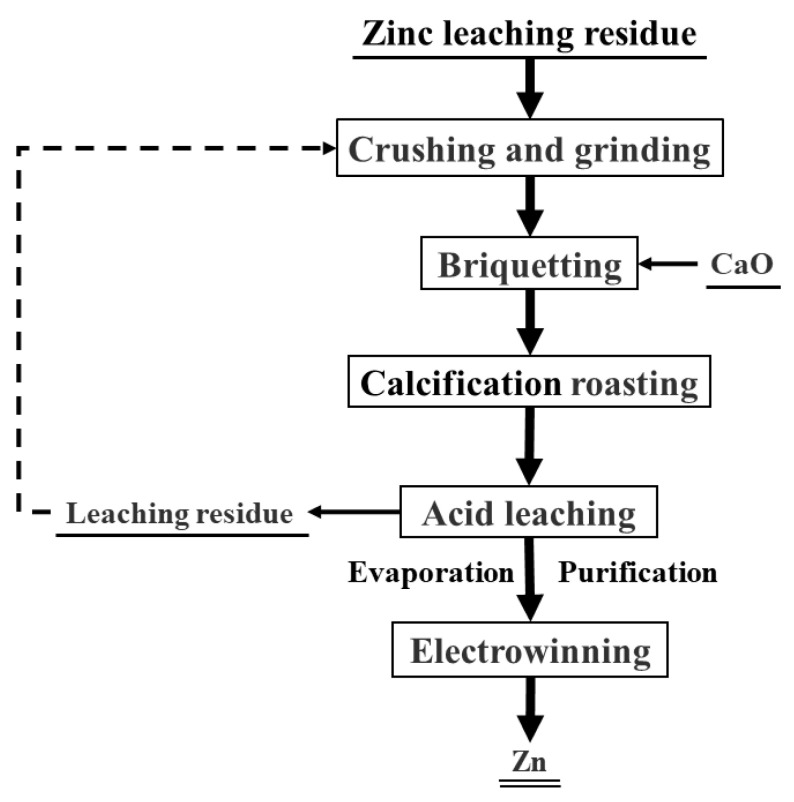
Flow chart of the whole process.

To increase the concentration of zinc in the electrolyte, the leachate should be evaporated. Electrowinning was then performed at ambient temperature for 60 min using 100 mL of evaporated solution. With reference to actual industrial production, lead anode and aluminum cathode, each measuring 3 cm × 3 cm, were utilized with an electrode spacing of 30 mm. The cathodic current density was set at 40 mA/cm^2^. The voltage of the electrolysis process was monitored and subsequently used to calculate the energy consumption per 1 kg of deposited zinc. Following electrowinning, the purity of the zinc product was determined. The flowchart of the whole process as shown in Figure 2. The current efficiency in the zinc electrowinning process was calculated according to Equation (4).

(4) $$H=\frac{M_{Zn}}{q\;\text{×}\;I\;\text{×}\;t\;}\;\text{×}\;100\%$$

where *η* is the current efficiency, %; *M_Zn_* is the mass of zinc generated, g; *q* is the electrochemical equivalent of metal zinc, 1.2195 g/(A·h); *I* is the electrical current, A; *t* is the electrowinning time, h.

### 2.3. Analysis

The ZLR and products obtained from experiments were analyzed and evaluated. The particle size of ZLR was measured using a laser particle size analyzer (BT-9300ST, Bettersize Instruments, Dandong, China). The chemical compositions of the samples were determined using inductively coupled plasma–atomic emission spectroscopy (ICP-AES, PerkinElmer Optima-4300DV, PerkinElmer, Shelton, CT, USA). The crystal phases of ZLR and roasting residue were characterized using XRD (Bruker D8 Advance, Bruker AXS Corporation, Ettlingen, Germany) under the operating conditions of 10–90° 2-theta and 8°/min with Cu Kα radiation at 40 kV and 40 mA. The micro-morphologies and element distributions were analyzed using SEM along with an EDS spectrometer (Zeiss Gemini SEM 300, Carl Zeiss AG Corporation, Oberkochen, Germany). A constant-current electrolysis experiment was performed using the Neware Battery Test System (Shenzhen Neware Technology Co., Ltd., Shenzhen, China).

## 3. Results and Discussion

### 3.1. Thermodynamic Analysis

**Figure 3:**
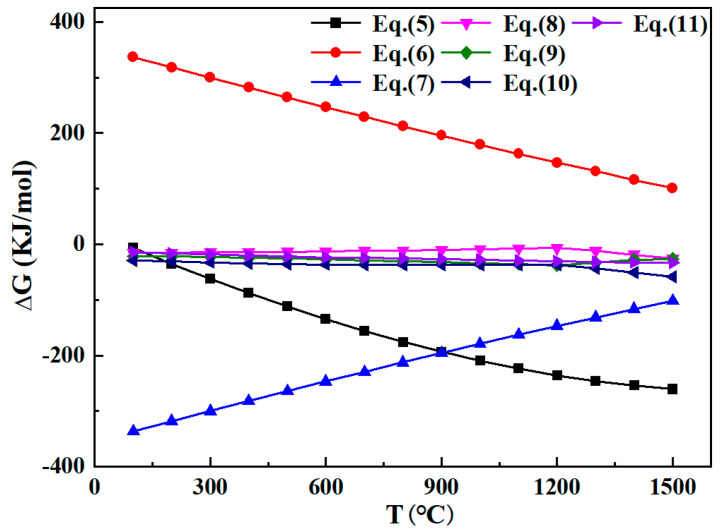
Trend of Gibbs free energy of all reactions with temperature.

The thermodynamic feasibility of calcination roasting of ZLR in the temperature range of 100–1500 °C was investigated using the HSC 6.0 database. Table 1 presents the possible reactions that occur during the roasting process of ZLR with CaO. The changes in reaction Gibbs free energy (ΔG^Ɵ^) for these reactions are illustrated in Figure 3.

The ΔG^Ɵ^ values of Equation (5) became significantly negative as the temperature increased, while Equation (6) was positive between 100 °C and 1100 °C. This indicates that CaSO_4_·2H_2_O only removed the bound water and did not undergo desulfurization reactions at 100–1100 °C. On the other hand, the ΔG^Ɵ^ of Equations (8)–(11) was negative at 100–1500 °C, suggesting that these reactions were thermodynamically feasible within that temperature range. It is worth noting that the calcification roasting process should be studied below 1200 °C, as that was found to be the melting temperature of Ca_2_Fe_2_O_5_ [24]. The reaction between ZnFe_2_O_4_ and CaO involves two reactions (Equations (8) and (9)), and the ΔG^Ɵ^ for Equation (9) was more negative compared to Equation (8). Therefore, it was more likely that the stable Ca_2_Fe_2_O_5_ would be produced during calcination roasting. Previous investigations have shown that some zinc ferrite was produced during the roasting process [25].

### 3.2. Calcification Roasting

**Figure 4:**
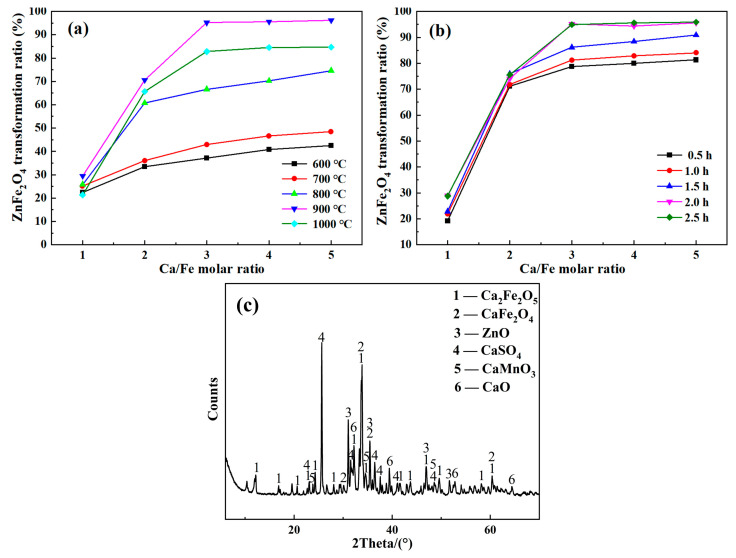
Effects of (**a**) roasting temperature and Ca/Fe molar ratio on Zn extraction (roasting time: 2 h); (**b**) roasting time and Ca/Fe molar ratio on Zn extraction (roasting temperature: 900 °C), (leaching condition: L/S of 30:1, 60 °C, 90 min, 100 g/L H_2_SO_4_); (**c**) XRD pattern of the roasted product (roasting condition: 2 h, 900 °C, Ca/Fe molar ratio of 3).

The transformation of ZnFe_2_O_4_ in ZLR was directly influenced by the roasting temperature, roasting time, and Ca/Fe molar ratio. To investigate the effects of roasting temperature and Ca/Fe molar ratio on the transformation of ZnFe_2_O_4_, the roasting time was kept constant at 2 h. The results are shown in Figure 4a. It was observed that as the roasting temperature varied between 700 and 900 °C, the transformation ratio increased rapidly with the increase in temperature. In terms of reaction kinetics, increasing the temperature enables more molecules to become activated, thus speeding up the reaction rate. It also increases the chances of a chemical reaction between calcium oxide and zinc ferrite, which in turn leads to a more complete reaction. However, when the roasting temperature exceeded 900 °C, the transformation ratio decreased significantly. Figure 4b indicates that at higher temperatures and lower Ca/Fe molar ratios, Equation (11) was more likely to occur, leading to the formation of additional ZnFe_2_O_4_ [24]. Therefore, a roasting temperature of 900 °C was deemed suitable.

The effects of roasting time and Ca/Fe molar ratio on the transformation of ZnFe_2_O_4_ were investigated, with the roasting temperature kept at 900 °C. As depicted in Figure 4b, the curves demonstrate that the transformation ratio gradually increases with the increase in roasting time from 0.5 h to 2.0 h. Afterward, it stabilizes with further increments in roasting time. This suggests that the reaction between zinc ferrite and calcium oxide during roasting reached equilibrium after 2 h. Overall, a roasting time of 2 h was considered the most appropriate.

According to Figure 4, the transformation ratio of ZnFe_2_O_4_ showed a significant increase as the Ca/Fe molar ratio increased from 1 to 3. However, further increasing the Ca/Fe molar ratio did not affect the transformation ratio. This suggests that a moderate amount of CaO was necessary to facilitate the contact between ZnFe_2_O_4_ and CaO in ZLR. In summary, the most suitable roasting conditions were found to be a Ca/Fe molar ratio of 3, a roasting temperature of 900 °C, and a roasting time of 2 h. Under these conditions, the transformation ratio of ZnFe_2_O_4_ reached 95.27%.

The XRD pattern of the roasted samples at 900 °C for 2 h with a Ca/Fe molar ratio of 3 is shown in Figure 4c. The main components observed in the roasted product were Ca_2_Fe_2_O_5_, CaFe_2_O_4_, ZnO, and CaSO_4_. These findings were consistent with the results obtained from thermodynamic analysis. Additionally, the roasting process leads to the formation of CaMnO_3_ as the manganese oxides in the ZLR react with calcium oxide.

### 3.3. Acid Leaching

**Figure 5:**
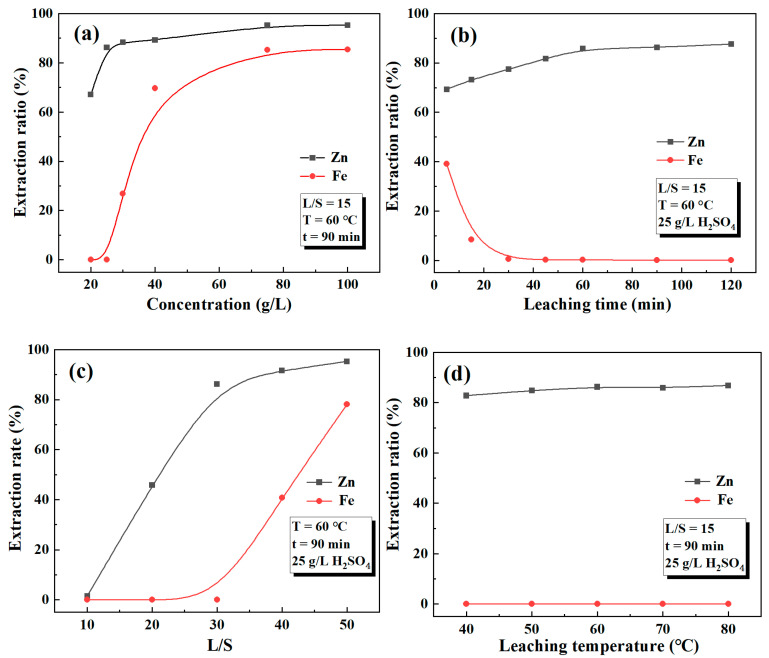
Effects of (**a**) acid concentration; (**b**) leaching time; (**c**) liquid-to-solid ratio; (**d**) leaching temperature on the extraction efficiencies of zinc and iron.

To effectively separate Zn and Fe in the roasted products obtained under optimal roasting conditions, we systematically investigated the effect of leaching conditions on Zn and Fe extraction, as shown in Figure 5. From Figure 5a, it is evident that the extraction efficiencies of Zn and Fe increased with the increase in acid concentration. As the acid concentration increased, there were more opportunities for collisions between hydrogen ions and reactant molecules, thus increasing the rate of reaction. However, the extraction efficiency of Fe showed a more significant increase. The difference between the extraction efficiencies of Zn and Fe was maximized at an acid concentration of 25 g/L. At this concentration, the extraction efficiencies of Zn and Fe were 86.26% and 0.06%, respectively. Therefore, a concentration of 25 g/L was chosen to achieve the separation of Zn and Fe.

**Figure 6:**
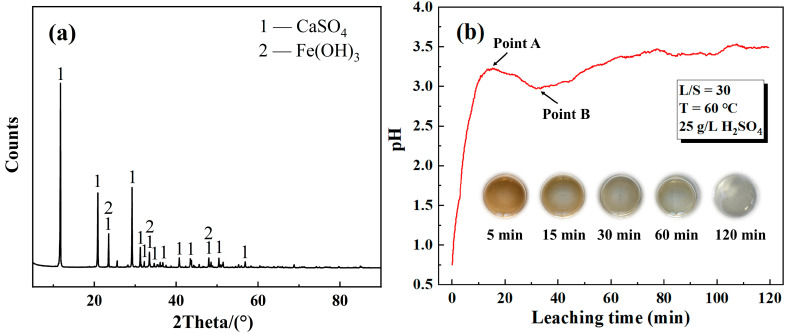
(**a**) XRD of leaching residue, and (**b**) pH change during leaching process (leaching condition: L/S of 15, 60 °C, 90 min, 25 g/L H_2_SO_4_).

Figure 5b illustrates the extraction efficiencies of Zn and Fe over different leaching times. It was observed that the extraction efficiency of Zn consistently increased, while that of Fe decreased as the leaching time was prolonged. The extraction of Zn and Fe reached a plateau after 90 min of leaching. The pH variation in the leachate concerning leaching time is depicted in Figure 6b. Before point A, the dissolution reaction of ZnO, Ca_2_Fe_2_O_5_, and CaFe_2_O_4_ dominated, resulting in an increasing pH value due to continuous acid consumption. Between points A and B, a decreasing trend in pH was observed, which can be attributed to the hydrolysis of iron ions in the leaching solution. When point B was reached, the extraction efficiency of iron decreased to 0.6%, and a majority of the iron ions in the solution hydrolyzed to form Fe(OH)_3_. Subsequently, the pH continued to increase and eventually stabilized. Moreover, with the extension of leaching time, the leaching solution gradually changed from yellow to colorless due to the decrease in iron ions. The relevant chemical reactions occurring during the leaching process were as follows:

ZnO + H_2_SO_4_ = ZnSO_4_ + H_2_O (12)

Ca_2_Fe_2_O_5_ + 5H_2_SO_4_ = 2CaSO_4_ + Fe_2_(SO_4_)_3_ + 5H_2_O (13)

CaFe_2_O_4_ + 4H_2_SO_4_ = CaSO_4_ + Fe_2_(SO_4_)_3_ + 4H_2_O (14)

Fe_2_(SO_4_)_3_ + 6H_2_O = 2Fe(OH)_3_ + 3H_2_SO_4_ (15)

The effect of L/S on the extraction ratios of zinc and iron at a leaching temperature of 60 °C, a leaching time of 90 min, and an acid concentration of 25 g/L is shown in Figure 5c. As the L/S ratio increased, the extraction ratios of Zn and Fe also increased. The increase in the L/S ratio makes the concentration of the slurry decrease, and the diffusion conditions of the leaching agent to the surface of the ore particles were improved, thus increasing the leaching rate of the metals. The maximum difference between Zn and Fe extraction was observed when the L/S ratio was 15, and the extraction ratio of Fe increased significantly with a further increase in the L/S ratio. Therefore, a liquid-to-solid ratio of 15 was chosen as appropriate for the selective extraction of zinc. The effect of leaching temperature on the extraction of Zn and Fe is illustrated in Figure 5d. It can be observed that the impact of leaching temperature on the extraction efficiency was minimal. The extraction efficiency of Zn showed a slight increase and remained stable beyond 60 °C. Considering the economic aspect, a lower temperature was more cost-effective. Hence, the leaching temperature of 60 °C was deemed suitable.

Subsequently, the leaching residue obtained under the optimized leaching conditions was analyzed by XRD, as depicted in Figure 6a. The XRD pattern revealed that the leaching residue primarily consisted of CaSO_4_ and Fe(OH)_3_, which was in agreement with the previous analysis results. Leaching residues can be used for cement production after simple roasting and dewatering. The chemical composition of the leachate obtained under the aforementioned optimized leaching conditions is presented in Table 2.

### 3.4. Residue Composition and Characterization

**Figure 7:**
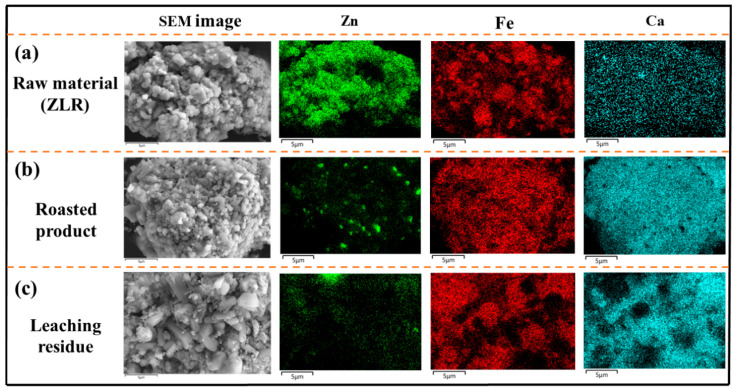
SEM and EDS analyses of raw material (ZLR), roasted product, and leaching residue (roasting condition: 2 h, 900 °C, Ca/Fe molar ratio of 3; leaching condition: L/S of 15, 60 °C, 90 min, 25 g/L H_2_SO_4_).

To investigate the transformation of phases and compositions of various solid residues generated throughout the process, SEM-EDS analyses were conducted on the raw material (ZLR), roasted product, and leaching residue, as depicted in Figure 7. The solid residues consist of irregular aggregates of particles. The EDS results revealed a partial overlap in the distribution of Zn and Fe in the ZLR, while Ca was uniformly distributed. The particles with high levels of Zn and Fe correspond to zinc ferrite, whereas the non-overlapping portions represent zinc sulfate and iron oxide, respectively. The uniformly distributed Ca in the ZLR exists in the form of calcium sulfate. These findings were consistent with the analysis presented in Figure 1a.

As depicted in Figure 7b, the roasted product exhibits a uniform and highly overlapped distribution of Fe and Ca, whereas Zn was independently distributed. This can be attributed to the formation of calcium ferrite and zinc oxide during calcification roasting. Upon acid leaching, the majority of Zn and a small fraction of Fe in the roasted product dissolve, leaving behind undissolved Ca_2_Fe_2_O_5_ and CaSO_4_ in the leaching residue. Figure 7c illustrates the distribution of Ca and Fe in the leaching residue, revealing that the striated particles correspond to CaSO_4_, while the spherical particles correspond to Ca_2_Fe_2_O_5_.

Table 3 presents the chemical composition of ZLR, roasted product, and leaching residue. The inclusion of CaO in the roasted product led to a decrease in the content of other components, except for Ca. It is worth noting that the total amount of components remained unchanged after calcification roasting, suggesting that no dust was generated during this process. Subsequently, during the leaching process, the ZnO in the roasted product dissolved, resulting in a decrease in Zn content to 1.23% in the leaching residue.

### 3.5. Electrowinning

**Figure 8:**
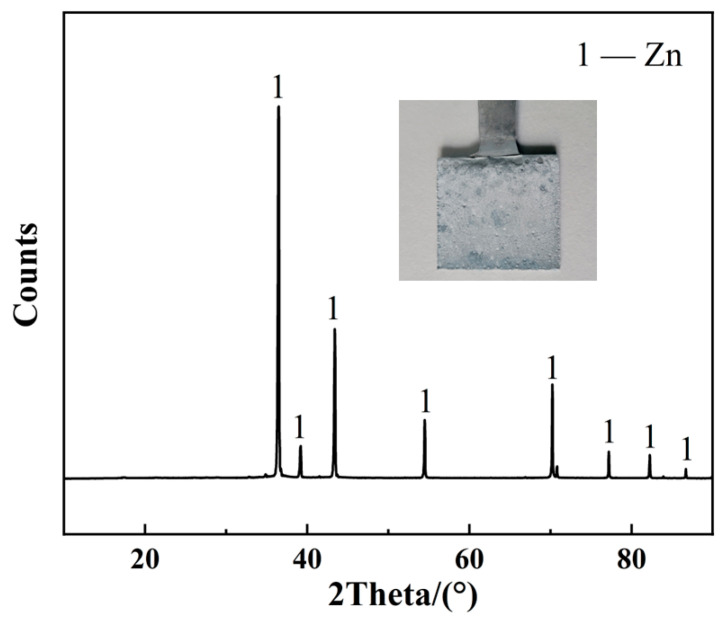
Naked-eye view and XRD pattern of the electrowinning product.

The chemical composition of the leachate obtained under optimal leaching conditions after evaporation treatment is provided in Table 4. The impurity elements in the leachate meet the standard requirements of the conventional zinc hydrometallurgy process. Electrowinning was conducted at a constant current density of 40 mA/cm^2^ for 60 min. The XRD pattern in Figure 8 illustrates the precipitated products on the cathode surface, which consist of silver-white metal zinc with a purity of 99.53%. Furthermore, chemical analyses and calculations showed that the current efficiency and energy consumption were 86.7% and 5.1 kWh/kg, respectively. The approximate cost of this process is primarily composed of raw material costs (calcium oxide and sulfuric acid) and production costs (roasting, leaching, and evaporation). A rough estimate indicates that the cost per ton of zinc produced through this process is approximately USD 2592.52. According to publicly available information, the average price of zinc metal in China’s domestic spot market was projected to be approximately USD 3214.72 per ton in 2024. This indicates that the process is economically viable.

The electrowinning process for producing metal zinc occurs via Equations (16) and (17). In the presence of an electric current, ZnSO_4_ in the electrolyte decomposes, and zinc metal precipitates on the cathode surface. The SO_4_^2−^ ions react with the H^+^ ions generated from the water decomposition, forming a sulfuric acid solution, while oxygen was released at the anode. The electrode reactions can be represented by Equations (18) and (19).

ZnSO_4_ + H_2_O = Zn(s) + H_2_SO_4_ + 1/2O_2_(g) (16)

ZnSO_4_ → Zn^2+^ + SO_4_^2−^ (17)

Cathode: Zn^2+^ + 2e^−^ → Zn(s) (18)

Anode: H_2_O − 2e^−^ → 1/2O_2_(g) + 2H^+^ (19)

## 4. Conclusions

An efficient calcification roasting–acid leaching process was proposed for the selective extraction of Zn and recovery of metallic zinc from the leaching residue of the traditional zinc hydrometallurgy process. In this work, we investigated the effects of the roasting parameters on the roasting process. The presence of calcium oxide enables the conversion of insoluble ZnFe_2_O_4_ in ZLR to ZnO, Ca_2_Fe_2_O_5_, and CaFe_2_O_4_, which facilitates the efficient recovery of Zn in the subsequent acid leaching process. The optimal roasting conditions were determined as a Ca/Fe molar ratio of 3, a roasting temperature of 900 °C, and a roasting time of 2 h. Under these conditions, the transformation ratio of ZnFe_2_O_4_ was found to be 95.27%. Following the roasting step, Zn was selectively extracted from the roasted product through acid leaching using a 25 g/L H_2_SO_4_ solution at 60 °C for 90 min, with an L/S ratio of 15. The extraction efficiencies of Zn and Fe were measured as 86.26% and 0.06%, respectively. Finally, a purity of 99.53% zinc metal was obtained through constant-current electrolysis for 60 min, with a current efficiency of 86.7%. This work demonstrates the feasibility of using calcium oxide as an additive in the treatment of ZLR with selective recovery of zinc.

## Figures and Tables

**Table 1:** The main chemical reactions occurring during the calcification roasting process.

Reaction	Equations
CaSO_4_·2H_2_O = CaSO_4_ + 2H_2_O(g)	(5)
CaSO_4_ = CaO + SO_3_(g)	(6)
CaO + SO_3_(g) = CaSO_4_	(7)
ZnFe_2_O_4_ + CaO = CaFe_2_O_4_ + ZnO	(8)
CaFe_2_O_4_ + CaO = Ca_2_Fe_2_O_5_	(9)
CaO + Fe_2_O_3_ = CaFe_2_O_4_	(10)
ZnO + Fe_2_O_3_ = ZnFe_2_O_4_	(11)

**Table 2:** Chemical composition of the leachate (leaching condition: L/S of 15, 60 °C, 90 min, 25 g/L H_2_SO_4_).

Element	Fe	Zn	Mn	Pb	Cu	Ca
Concentration (mg/L)	1.84	8.01 × 10^3^	7.10	<0.01	0.01	13.02

**Table 3:** Chemical composition of ZLR, roasted product, and leaching residue (roasting condition: 2 h, 900 °C, Ca/Fe molar ratio of 3; leaching condition: L/S of 15, 60 °C, 90 min, 25 g/L H_2_SO_4_).

Components (wt. %)	Fe	Zn	Mn	Pb	Cu	Ca	S
Zinc leaching residue	21.67	16.20	7.29	1.82	0.43	0.91	7.30
Roasted product	13.63	10.30	3.68	1.06	0.30	28.49	4.70
Leaching residue	9.19	1.23	2.45	0.69	0.01	17.35	15.20

**Table 4:** Chemical composition of the solution after evaporation treatment and cathodic zinc products.

Element	Zn	Fe	Cu	Pb	Ca	Mn
Content (mg/L)	80.4 × 10^3^	9.2	0.07	0.01	53	720
Content (wt. %)	99.53	0.01	0.2	0.12	\	\

## Data Availability

The original contributions presented in this study are included in the article. Further inquiries can be directed to the corresponding author.
